# Severe fetomaternal transfusion due to an unrecognized chorangioma in a dichorionic, diamniotic twin pregnancy

**DOI:** 10.1007/s00404-025-08249-3

**Published:** 2026-01-13

**Authors:** Jonas Bubmann, Christian Dannecker, Manuela Franitza, Marina Seefried, Philipp Voisard, Udo Jeschke, Carl Mathis Wild, Fabian Garrido, Tina Schaller, Bernadette Eser

**Affiliations:** 1 Department of Gynecology & Obstetrics, Faculty of Medicine, University of Augsburg, Stenglinstrasse 2, 86156 Augsburg, Germany; 2 Department of Pathology, Faculty of Medicine, University of Augsburg, Stenglinstrasse 2, 86156 Augsburg, Germany

**Keywords:** Fetomaternal transfusion, Chorangioma, Twin pregnancy, Fetal hemoglobin

## Abstract

**Objectives/hypothesis:**

Severe fetomaternal transfusion due to an unrecognized chorangioma in a dichorionic, diamniotic twin pregnancy: case report and review of the literature.

**Study design:**

Case report and retrospective narrative review.

**Methods:**

Evaluation of 8 case reports.

**Case report:**

A 32-year-old primigravida with dichorionic, diamniotic twin pregnancy developed growth discordance and hypertension. At 35 weeks, cesarean delivery revealed one viable twin and one severely anemic twin who died immediately postnatal. Fetomaternal transfusion was suspected, confirmed, and most likely caused by a 6 cm chorangioma.

**Conclusion:**

Fetomaternal hemorrhage is a serious but underrecognized complication and can be caused of placental chorioangiomas, among other things. Although rare, it poses significant risks of fetal anemia and perinatal morbidity. Increased awareness and routine Doppler monitoring of at-risk fetuses may facilitate earlier diagnosis and timely intervention, potentially improving outcomes. Particularly in children with anemia, fetomaternal transfusion should always be considered and HbF material determined.

## Introduction

Fetomaternal transfusion (FMT), also termed massive fetomaternal hemorrhage (FMH), denotes the pathological transfer of fetal erythrocytes into the maternal circulation, typically involving more than 30 mL of fetal blood [1]. Although small-volume fetomaternal hemorrhage (FMH) is a physiological phenomenon occurring in the majority of pregnancies, clinically significant or massive FMH represents a rare but serious obstetric complication, with an estimated incidence of 1 in 3000 to 1 in 10,000 live births [2]. Clinical manifestations may include reduced fetal movements, non-reassuring cardiotocography, fetal hydrops, or intrauterine fetal demise. Diagnostic confirmation relies on quantitative methods such as the Kleihauer–Betke acid elution test or flow cytometry, which allow measurement of fetal erythrocytes within the maternal circulation [3]. Despite advances in perinatal diagnostics, FMH remains frequently underrecognized due to its often subtle and nonspecific clinical presentation.

Chorangiomas are non-trophoblastic vascular tumors of the placenta characterized by the proliferation of capillary vessels within chorionic villi [4]. They represent the most common type of placental tumor, with an incidence of approximately 0.5–1% in histopathological series [5]. While most chorangiomas are small and clinically insignificant, lesions exceeding 4–5 cm in diameter—commonly referred to as “giant chorangiomas”—have been associated with adverse perinatal outcomes, including polyhydramnios, fetal anemia, hydrops fetalis, intrauterine growth restriction, and stillbirth [6]. These complications are thought to arise from arteriovenous shunting within the tumor, leading to fetal cardiac overload, hemolysis, and, in some cases, hemorrhage into the maternal circulation [7].

Although several case reports have suggested a possible association between large chorangiomas and FMH, the underlying mechanisms, true incidence, and clinical relevance of this relationship remain insufficiently understood. The objective of this systematic review is to summarize the available evidence on the interplay between chorangiomas and fetomaternal transfusion, and to assess the implications for diagnosis, perinatal management, and neonatal outcomes.

## Case presentation

The patient has given her written consent to publication. A 32-year-old primigravida with a spontaneously conceived dichorionic–diamniotic twin pregnancy after ovulation induction was referred at 20 weeks for routine anomaly screening. Both male fetuses appeared structurally normal, with an initial weight discordance of 12.5%. Growth and Doppler evaluations at 22, 24, and 28 weeks showed appropriate interval growth and stable discordance.

At 30 + 0 weeks, the patient was hospitalized for cervical insufficiency (7 mm), and antenatal corticosteroids and tocolysis were administered. A significant increase in growth discordance (25%) was noted. She remained inpatient for 4 weeks due to newly diagnosed pregnancy-induced hypertension (PIH), managed with Methyldopa and Nifedipine. At 33 + 5 weeks, she was discharged at her own request despite progressive growth discordance (32%) and persistent hypertension under treatment.

At 34 + 6 weeks, outpatient sonography showed difficulty assessing the second twin due to fetal position and maternal habitus. Growth discordance was noted at 15%, with normal Doppler indices. Blood pressure remained poorly controlled despite triple antihypertensive therapy (Methyldopa, Nifedipine, Metoprolol). The patient refused the recommended inpatient admission.

At 35 + 1 weeks, during the panned checkup, a hypertensive crisis (165/125 mmHg) prompted immediate hospitalization, initiation of intravenous Urapidil and magnesium sulfate, and cesarean section. Doppler ultrasound of both fetuses particularly middle cerebral artery peak systolic velocity (MCA-PSV) of the second twin appeared normal at the time of the surgical indication. For reasons of operating capacity, the cesarean section was performed more than 3 h later under spinal anesthesia. In the meantime, CTG monitoring took place, whereby the second Geminus could not be derived due to the position of the baby and maternal obesity (BMI 36), which was not new, however, as it was known from the previous examinations. The leading twin was delivered in good condition (APGAR: 9/10/10, pH: 7.24); however, the second fetus appeared pale and lifeless at delivery (APGAR: 0/0/0). The initial blood gas analysis revealed a hemoglobin level of 2.2 g/dL, prompting emergency transfusion during ongoing resuscitation. Despite more than 30 min of resuscitation efforts, the infant died.

The suspicion of a fetomaternal transfusion was confirmed by analysis of maternal blood. In the maternal circulation, *2.46%* fetal erythrocytes were detected by flow cytometry, corresponding to an estimated fetal blood loss of approximately 123 mL.

**Fig. 1:**
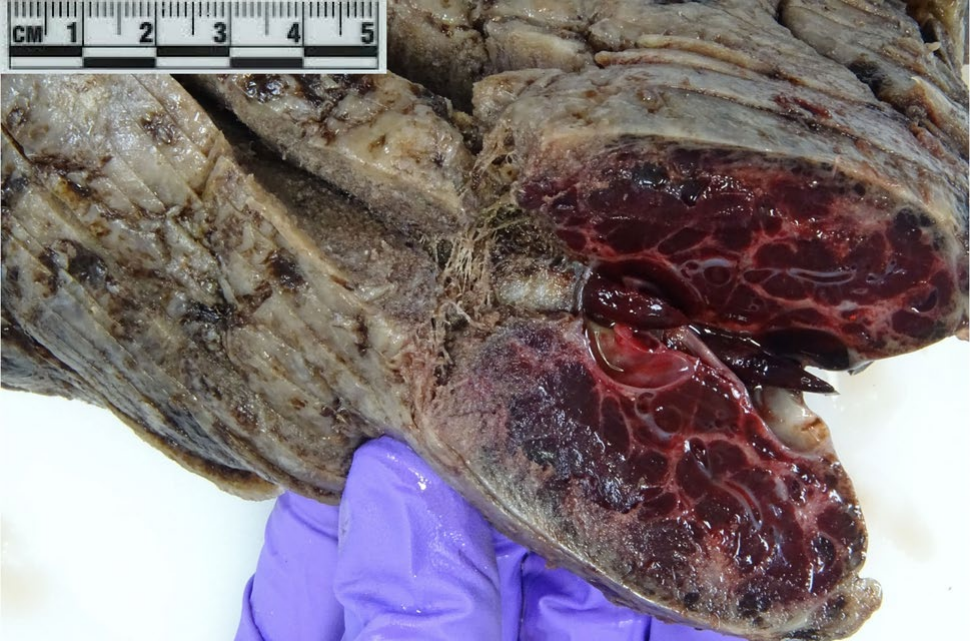
Macroscopic picture of the chorangioma

When examining the placenta of the deceased twin, a hard and fibrotic looking area of approximately 5 cm was noticed (see Fig. 1). Histopathological examination of the placenta revealed a previously undiagnosed 6 cm chorangioma, which is consistent with an acute manifestation of fetomaternal transfusion (see Fig. 3a and b).

Unfortunately, the chorangioma was not detected by prepartum sonography, most likely due to the difficult sonographic conditions with maternal obesity, fetal position and posterior wall placenta. Even with the retrospective assessment of the ultrasound images from the 20th to the 36th week with the knowledge of the presence of the chorangioma, no sonographic correlate could be found.

## Review of literature

### Materials and methods

In July 2025, the search was carried out with various search terms in *Pubmed*®. The initial search was for case reports and case descriptions of fetomaternal transfusion with chorangiomas. The data collection was carried out by two authors (J. B., B. E.).

**Fig. 2:**
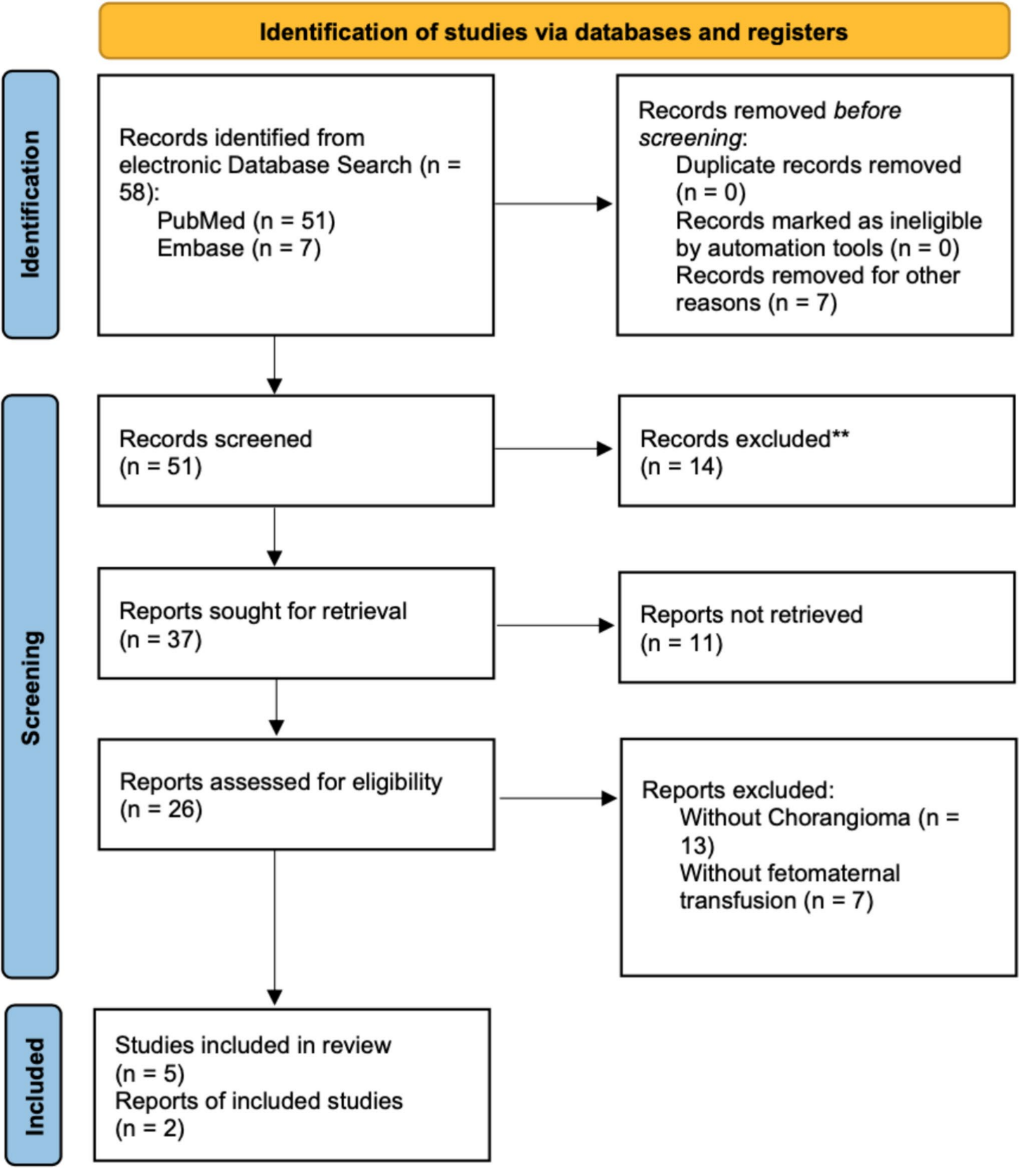
PRISMA 2020 flow diagram for new systematic reviews

*58* cases were found and *51* of them further screened. *26* cases had been eligible, but only *7* of them had fetomaternal hemorrhage associated with a chorangioma (see Fig. 2). All cases before the introduction of ultrasound diagnostics were excluded. The oldest included case was from 1976 [8], four cases were before 2000 [8–11], no case between 2000 and 2010 and 3 cases since 2010 to date [12, 13]. The most recent case was from 2022. One study was translated from Spanish [10]. The remaining cases were in English. We include our above-mentioned case in the systematic review.

A systematic evaluation of reported cases of FMH associated with placental chorioangiomas was performed using data extracted from published case reports. Information analyzed includes gestational age at diagnosis and delivery, tumor size, fetal hemoglobin levels, evidence of hydrops, perinatal interventions, birth outcomes, and maternal–fetal blood group compatibility. The data were collected manually by the first author.

### Results

A total of 7 case reports detailing fetomaternal transfusion associated with placental chorioangiomas were identified, with publication years ranging from 1976 to 2022. The gestational age at diagnosis varied between 32 and 39 weeks *(m = 36.88; ± 1.45; n = 8)*. Most cases were diagnosed in the third trimester, with delivery typically occurring between 35 and 39 weeks of gestation [8, 12, 13].

Tumor sizes ranged from as small as 1.0 cm [13] to as large as 6 cm (our case). The average tumor size was *m* (mean) = 2.85 cm (± 1.94 cm; *n* = 7), suggesting that tumor vascularity may play a more critical role than absolute size [12, 13]. Larger tumors were more commonly reported in earlier publications [8, 9].

Severe fetal anemia was a consistent finding across the cases, with median hemoglobin levels of *5.82 g/dL (*± *2.80; n* = *8)* reported at birth. Fetal hemoglobin (HbF) values were significantly elevated on average at *3.82% (*± *1.96; n* = *8)*, further confirming the diagnosis of FMH and highlighting the magnitude of fetal blood loss into the maternal circulation. Only one case has reported a pathological middle cerebral artery peak systolic velocity (MCA-PSV) measurement where values had been elevated, supporting the presence of fetal anemia due to ongoing hemorrhage [12].

Despite the severity of fetal anemia, most neonates survived, though many *(n* = *6)* required postnatal blood transfusions. Unfortunately, two newborns died despite receiving transfusions. In these two cases, relatively low HbF levels and different hemoglobin levels are evident in comparison.

Not a single patient received intrauterine blood transfusions. The average placental weight was *889 g (*± *979 g; n* = *7)*. The average age of the mothers was *30.0 years (*± *6.9; n* = *6)*. *83%* of the reported patients were primigravida *(n* = *6). 75% of the children were female.*

Maternal–fetal blood group analysis showed no consistent pattern of Rh or ABO incompatibility, suggesting that the FMH was not immune-mediated but rather mechanical in nature due to tumor-associated vascular shunting or rupture [9, 13]. All reported patients *(n* = *5)* and children *(n* = *6)* were Rhesus positive. APGAR scores at 1 and 5 min ranged from low (1/1) to moderate (7/8), reflecting varying degrees of neonatal compromise at delivery.

Delivery was mostly via cesarean section, 3 of which were performed as emergency cesarean sections. A spontaneous delivery was reported, in which case the diagnosis of fetomaternal transfusion was made postpartum. [8]. All reported children received intensive postpartum care (*n* = 7, summarized in Table 1 together with present case).

**Table 1:** Fetomaternal transfusion cases found in the literature together with current case associated with placental chorioangiomas

Year	Author	Week of diagnosis	Week of delivery	Tumor size (cm)	Hb (g/dL)	HbF (g/dL)	Suspicious MCA	Hydrops-signs	Placental weight	Intrauterine transfusion	Mode of delivery	Postnatal ICU	Outcome	Postpartum transfusion	Age of mother (years)	Previous pregnancy	Birthweight (g)	APGAR	Sex	BG mother	BG child
2012	Kawano et al	36	36	1.50	4.30	4.60	Yes	No	450	No	Emergency cesarean section	Yes	Healthy	Yes	37	Primipara	1840	5/5	0	No data	No data
2022	Kinoshita	35	35	1.30	9.90	2.10	No data	No	700	No	Emergency cesarean section	Yes	Healthy	No	No data	1 × Cesarean section	2596	7/8	0	B+	A+
2022	Kinoishita	37	37	1.00	4.00	5.10	No data	No	520	No	Emergency cesarean section	Yes	Healthy	Yes	No data	No data	2418	5/5	0	B+	B+
1976	Sims et al	32	39	2.20	8.40	7.00	No data	No	3100	No	Vaginal	Yes	Health	No	32	Primipara	2200	1/1	1	A+	A+
1986	Stiller et al	39	39	5.00	7.50	1.50	No data	No	550	No	Cesarean section	No data	Died	No data	36	No data	No data		0	A+	0+
1987	Santamaria et a	36	36	No data	7.30	2.40	No data	No data	453	No	Cesarean section	Yes	Healthy	Yes	23	Primipara	2425	1/1	0	No data	No data
1989	Brandt et al	37	37	3.00	3.00	5.40	No data	No	No data	No	Cesarean section	Yes	Died	Yes	20	Primipara	2340	1/4	0	No data	AB+
2024	Bubmann et al	36	36	6.00	2.20	2.46	No	No	450	No	Cesarean section	No	Died	Yes	32	Primipara	1600	0/0	1	A+	+

## Discussion

**Fig. 3:**
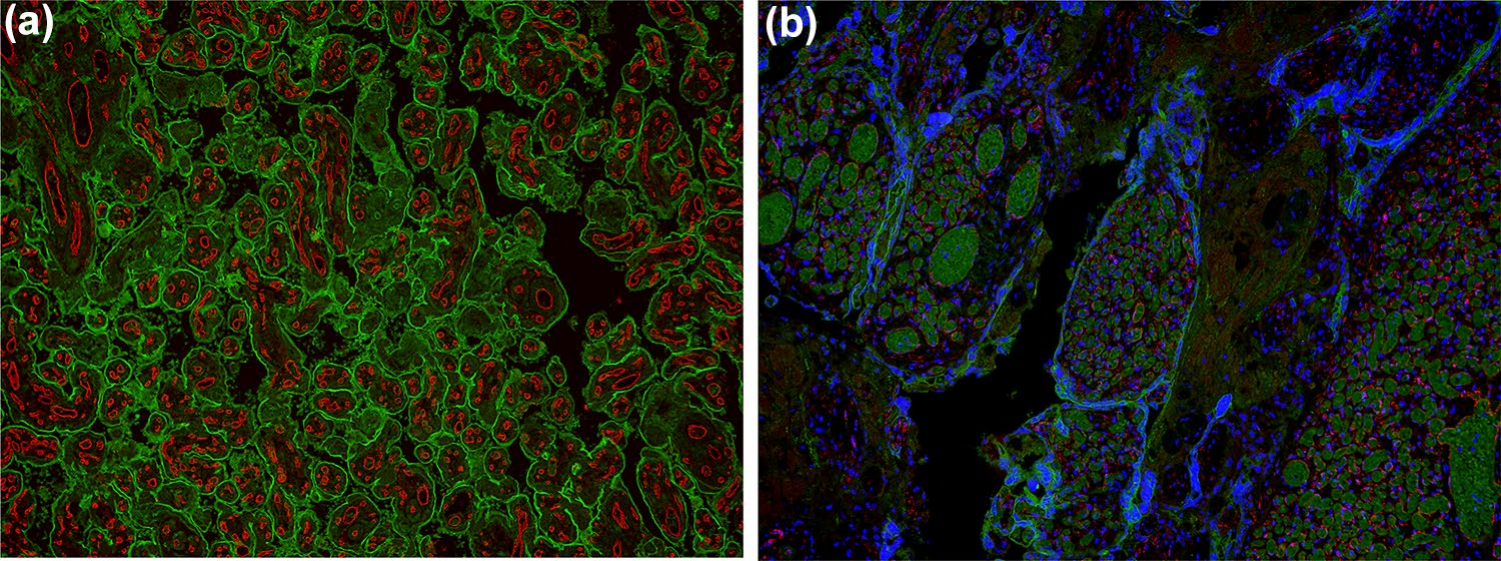
**a** Placenta of the healthy twin without chorangioma shows PDL-1-staining at the syncytiotrophoblast in green and CD31 staining of the fetal vessels inside placental villi in red with no microscopic evidence of a fetomaternal transfusion site. **b** Placenta of the twin with chorangioma shows disrupted PDL-1-staining at the syncytiotrophoblast in green and CD31 staining of the fetal vessels inside placental villi in red and cell nuclei in blue (DAPI) with microscopic evidence of a fetomaternal transfusion site. Striking finding of a secretory product with greenish self-fluorescence inside the fetal vessels with chorangioma

This review underscores the potential for substantial fetal morbidity in cases of acute fetomaternal hemorrhage (FMH) associated with placental chorioangiomas. The underlying pathophysiological mechanism is presumed to involve rupture or leakage from the highly vascular tumor into the maternal circulation, particularly when the lesions are large or exhibit pronounced perfusion. For the visualization of the syncytiotrophoblast rupture, we used PD-L1 an immune checkpoint molecule, known from different gynecologic cancer types as endometrial cancer [14], breast cancer [15] and vulvar cancer [16]. In the placenta, PD-L1 is uniformly expressed by the syncytiotrophoblast [17] as shown in Fig. 3a. PD-L1 staining was used in this study to show either an undisturbed syncytiotrophoblast layer (Fig. 3a, green fluorescence) or a ruptured layer of the syncytiotrophoblast as shown in Fig. 3b in the case of the chorangioma. In addition, PD-L1 is also expressed by placental macrophages [18].

CD31 on the other side is a marker for endothelial cells [19, 20] and specifically used to stain fetal or maternal vessels at the fetomaternal interphase [21, 22]. In this study, CD31 was used to visualize intact fetal vessels within placental villi as shown in Fig. 3a (red fluorescence) or leakage of fetal blood to the maternal circulation in the case of the chorangioma with evidence of a fetomaternal transfusion site as shown in Fig. 3b with incomplete CD31 vessel staining.

Most reported cases occurred in the late third trimester, thereby limiting opportunities for timely in utero intervention. The markedly reduced fetal hemoglobin concentrations and elevated HbF levels observed in these cases are consistent with substantial fetal blood loss into maternal circulation. In the single case where middle cerebral artery peak systolic velocity (MCA-PSV) was assessed, the value was elevated, underscoring its utility as a non-invasive marker for fetal anemia.

Although acute FMH remains an uncommon event, its occurrence in association with a placental chorioangioma warrants intensified fetal monitoring, particularly when the tumor is large or demonstrates significant vascularization. Doppler evaluation of MCA-PSV should be considered an essential component of surveillance in such pregnancies.

## Conclusion

Fetomaternal transfusion is a serious yet frequently underrecognized complication, and placental chorioangioma represents one potential etiological factor. Despite its rarity, this condition poses considerable risks for severe fetal anemia and perinatal morbidity. Heightened clinical awareness, together with routine Doppler surveillance in pregnancies complicated by chorioangioma, may support earlier detection and timely intervention, thereby improving fetal outcomes. In neonates presenting with profound anemia, FMH should always be considered, and fetal hemoglobin quantification is recommended. Moreover, macroscopic inspection of the placenta and subsequent histopathological examination may help identify contributing pathologies, including placental chorioangioma.

## Data Availability

No datasets were generated or analysed during the current study.
